# Measuring the experience of social trauma: Development and evaluation of the Social Devaluation Questionnaire

**DOI:** 10.1371/journal.pmen.0000450

**Published:** 2025-10-27

**Authors:** Frank Neuner, Sina Neldner, Finn Krüger, Alexander Saada, Maren Kibar, Rezhna Mohammed

**Affiliations:** Bielefeld University, Bielefeld, Germany; PLOS: Public Library of Science, UNITED KINGDOM OF GREAT BRITAIN AND NORTHERN IRELAND

## Abstract

Stressful events and conditions can affect mental health. However, the psychopathological consequences of adverse events depend on how individuals think and feel during these experiences. Current instruments assessing peritraumatic distress focus on dysfunctional appraisal types, negative emotions, and dissociative experiences with little or no attention to social emotions. To fill this gap, this study describes the development and initial psychometric evaluation of an instrument to assess the affective response to stressful or traumatic events that threaten a person’s social status. Based on theoretical assumptions about reactions to social stressors, we developed the Social Devaluation Questionnaire (SODEQ) to assess the proposed affective state of social devaluation, consisting of specific emotional, cognitive, behavioral, and physiological facets. We tested the SODEQ on data from four studies, three online surveys with convenience samples totaling about N = 1200 participants and one face-to-face survey with N = 511 Syrian refugees living in refugee camps in the Kurdistan Region of Iraq. In each of the studies, social devaluation was assessed using a specific index event, i.e., emotional abuse, peer victimization, abandonment, and post-migration stress as a refugee. We found evidence for the reliability of the instrument and the validity of both the instrument and the construct. Factor analyses indicated that items clustered into highly correlated components but supported treating social devaluation as a unified affect. In each study, the SODEQ score was associated with both the severity of the index event and indicators of psychopathology, and social devaluation partially or fully mediated the relationship between event severity and outcomes. Social devaluation appears to be a meaningful construct representing an affective response to an intense social stressor that contributes to psychopathology. The SODEQ seems to be a valuable tool for identifying mechanisms behind symptoms resulting from threats to one’s social integrity.

## Measuring the experience of social trauma: Development and psychometric evaluation of the Social Devaluation Questionnaire (SODEQ)

During and immediately after stressful and traumatic events, individuals experience a range of negative emotions such as fear, anger, helplessness, or horror [1]. In traumatic stress research, affective states that occur during traumatic events are referred to as *peri-traumatic experiences* [2,3], which include negative emotions as well as distinct cognitive processes and physiological states. Some of these peritraumatic experiences are so beyond everyday experience that they lack common colloquial expressions. For example, many survivors of traumatic events report anomalous dissociative experiences such as out-of-body experiences and depersonalization during traumatic events [4]. Several questionnaires have been developed for the assessment of different types of peri-traumatic experiences including fear, helplessness, disgust, sadness, and anger [3] but also abnormal trauma-related experiences such as tonic immobility [5], dissociation [6], mental defeat [7], and others [2,8]. Research on posttraumatic stress disorder has consistently documented that the quality and intensity of negative peritraumatic reactions are predictors of the long-term harm caused by traumatic events [1].

To date, research on peri-traumatic experiences has been focused on events that meet the trauma criterion as defined in the diagnostic manuals. Both, the DSM and the ICD restrict the diagnosis of posttraumatic stress disorder to events that involve a threat to a person’s physical or sexual integrity. However, a growing body of research and clinical observation [9–12] indicates that some of the most harmful events in terms of long-term mental health outcomes involve no direct threat to life or limb but instead threaten a person’s social integrity, and in particular, their social status. For example, a robust body of cross-sectional and longitudinal research has shown that emotional abuse [13,14] and peer victimization [15–18] contribute to mental disorders and that emotional types of maltreatment may even exceed the harmful effects of physical and sexual abuse in various types of outcomes [14,19,20].

However, while several well-established psychological theories provide explanations for the development of PTSD following a traumatic event, little is known about the mechanisms that produce harm following threats to social status. Following the Physical and Social Trauma (PAST) theory [12] we use the term *social trauma* for such events: highly aversive, often repeated experiences that endanger an individual’s position in the social hierarchy through acts such as humiliation, degradation, defeat, or persistent exclusion. While social trauma can co-occur with physical threats, it can also occur in their absence, and is better understood through a trans-diagnostic lens than through the narrow event definition of PTSD. Consistent with influential theories of PTSD, peri-traumatic responses are thought to be encoded in memory networks and may later be reactivated by trauma reminders [21–23]. The PAST framework [12] builds directly on these models by extending their logic from threats to physical integrity to threats to social integrity, thereby conceptualizing social trauma as a parallel process in which status loss, humiliation, and rejection can trigger comparable peri-traumatic states. From an evolutionary perspective, social species including humans rely on hierarchical organization to regulate access to resources and reproduction; acute loss of rank or status can thus trigger a biologically prepared defense program involving freezing, fight/flight, and—when defeat is inevitable—behavioral demobilization and submission [24–31]. This defensive state is here referred to as social devaluation: the subjective and embodied experience of being lowered in rank or worth in the eyes of others to the extent that one’s social value is undermined. It comprises a coordinated affective state spanning characteristic emotions (e.g., shame, humiliation), cognitive appraisals (e.g., defeat, damaged honor, loss of self-worth), behavioral impulses (e.g., blocked fight/flight tendencies, withdrawal), and physiological reactions (e.g., motor inhibition, blunted arousal). Although some of these facets have been examined separately in the literature on social defeat, humiliation, shame, exclusion, and social defeat [21–28], there is no established measure that integrates them into a single construct rooted in the biology of status loss.

In general, there are few validated scales that allow for the assessment of the experiences during events that threaten social status. A prominent exception is the Defeat Scale [32], which assesses the immediate reactions to a perceived loss of status. While the strength of this instrument lies in its detailed assessment of the appraisals of the situation, it does not aim to assess the full picture of the affective response, including emotional and physiological reactions. Our aim in developing the Social Devaluation Questionnaire (SODEQ) was to address this gap by capturing all four domains—affective, cognitive, behavioral, and physiological—through verbally accessible descriptions of each component. These items were derived from the evolutionary and neurobiological literature on status loss, ensuring biological grounding and likely cross-cultural validity [12].

The Social Devaluation Questionnaire (SODEQ) was developed to capture the proposed affective state of social devaluation in a self-report or interview format. The development of the SODEQ is based on the view of emotions as affective programs that have evolved to maintain and restore basic needs during environmental challenges [33–35]. In this perspective, depending on the nature of a threat, a specific emotion is activated by a stressor, enabling the organism to mount a defensive response. Emotions are defined as unique constellations of cognitive appraisals, physiological reactions and behavioral impulses [33]. Common emotions can be identified and verbally communicated through labels such as fear and anger, which are transferable across languages and cultures. However, more nuanced types of reactions often lack ubiquitous verbal identifications [36].

In this framework, we propose social devaluation as an affective state that lacks a colloquial verbal description. In order to assess this state, the SODEQ items consist of labels of related emotions (e.g., shame) but also of experiential descriptions of emotion components such as thoughts, physiological reactions and action tendencies that have been associated with devaluation. As understood by social rank theory [37], social devaluation is associated with submissive behavior toward conspecifics that results in a self-perception of inferiority. As social devaluation arises from a biological defense program that responds to loss of status, it involves the appraisal of being defeated or rejected by others to the extent that one’s self has been devaluated and the person has lost status. In addition, it includes the behavioral response tendency to withdraw or hide (as is common to the emotion of shame) or to display powerless anger including the inhibition of aggression, as it is characteristic of humiliation [30]. In the context of the PAST framework, social devaluation involves a reduction in active defense and thus a blunted physiological response.

The SODEQ claims to be applicable to the assessment of reactions to events and conditions that occur across specific contexts and durations. Since many types of traumatic events, most notably sexual violence and physical assault, involve humiliation and degradation, the SODEQ can also cover reactions to the degrading aspects of events that meet classic trauma criteria. In order to accommodate this wide range of event types, the SODEQ was developed with the input of clinical researchers and therapists with experience in processing memories of various types of emotional events through exposure therapy. This approach allowed for the inclusion of subjective reports of devaluation that occur during child abuse, bullying, sexual violence, and war experiences. This study reports on the development and the first psychometric evaluation of the SODEQ. We then tested the SODEQ in a series of surveys with respect to four different types of social stressors, i.e., emotional abuse, peer victimization, abandonment, and post-migration stress as a refugee.

*Emotional abus*e consists of an abusive pattern of verbal and nonverbal communication that includes various forms of humiliation, rejection, belittling, threatening or ridiculing [38]. Emotional abuse, especially in the form of verbal abuse [39], is strongly associated with psychopathology in adulthood [40], even when controlling for other types of maltreatment [14,19,20]. *Peer victimization* consists of repeated experiences in which an individual is the victim of relational, physical, or verbal aggression by peers [41] during childhood and adolescence. It involves exclusion, rejection, humiliation, and threats. Peer victimization is also related to psychopathology, in particular depression [42] and symptoms of social anxiety [14]. *Abandonment* refers to the state of being left by a significant person, for example a romantic partner. While any consensual or non-consensual way of separating from a partner can cause negative emotions such as sadness and grief and increase the risk of developing depression [43–45], being abandoned by an intimate partner appears to be particularly distressing. Individuals who have been rejected experience more rumination, depression, and loss of self-esteem than those who initiated the breakup [46]. Abandonment is also associated with rejection, can cause more intense distress than bereavement [45] and is a major risk factor for suicide events in adolescence [47]. Finally, *post-migration stress* refers to stressors that refugees typically encounter in exile [48,49], which contribute to mental health problems [49,50]. Post-migration stress includes both the stressors associated with the loss of social networks due to flight and the restrictions on basic human rights such as freedom of movement, the right to work, and the right to education that characterize the refugee experience [51]. Post-migration stress is strongly related to discrimination, that refers to the unequal treatment of individuals based on group-related characteristics such as race, sexual identity, or disability. The perception of being discriminated against [52,53], but also objective discrimination, is associated with poor mental health [54] which is in part related to the stress of exclusion, rejection and degradation [55]. In this study, we investigated the impact of post-migration stress among Syrian refugees living in refugee camps in the Kurdistan Region of Iraq (KRI).

The purpose of this study was to test the validity of the construct of social devaluation and to provide initial indicators of the psychometric quality of the SODEQ. If social devaluation is a meaningful construct, then the items of the SODEQ should be highly intercorrelated within each sample and load on a single factor, even though they assess indicators of a proposed affect in different domains (emotions, appraisals, physiological responses, and behavioral responses). This high intercorrelation can be tested by determining internal consistency, i.e. Cronbach’s alpha, complemented by a factor analysis. Second, if social devaluation assesses the affective response to a social stressor, it should be related to indicators of the severity of different types of social stressors, i.e., emotional abuse, peer victimization, separation, and post-migration stress. Third, according to the theoretical foundation of the concept, social devaluation should function as a mechanism that translates stress into psychopathology. Consequently, social devaluation, as measured by the SODEQ, should play a mediating role in the relationship between the severity of social stressors and indicators of mental health.

## Method

### Participants

This psychometric evaluation study used data from three online surveys, referred to here as the Peer Victimization Study, the Emotional Abuse Study, and the Breakup Study, and one in-person survey, referenced here as the Migration Study. While the Peer Victimization Study included a broad range of participants, the Breakup Study recruited only individuals who reported the breakup of an intimate relationship within the past 12 months. Because the original goal of the Emotional Abuse Study was to examine the relationship between childhood trauma and academic success, only university students were eligible to participate in this study.

Peer Victimization Study: Data for this study was collected between 26.02.2024 and 28.03.2024. Participants were recruited from the general community via social media and online platforms, as well as from a university student participation portal, resulting in a mixed sample of students and non-students. A total of 783 individuals began the study and 525 participants completed the questionnaire, of which 30 participants were excluded due to dropout. Data from five additional participants were excluded based on an outlier analysis using Cook’s distance, leaving a final sample of n = 490 participants aged 18–73 years (M = 30.6, SD = 11.0). A total of 78 males (16%), 407 females (83%), and five individuals who identified as non-binary participated. Participants had a mean of 12.4 years (SD = 2.5) of education, and 75% of participants reported being employed. Participants reported a mean value of peer victimization in the FBS of *M* = 11.81 (*SD* = 8.00), of social devaluation in the SODEQ of *M* = 18.12 (*SD* = 11.70), depression in the PHQ-9 of *M* = 8.79 (SD = 5.61) and social anxiety in the SPIN of *M* = 21.14 (*SD* = 13.40).

Emotional Abuse Study: This study was online between 15.05.2024 and 20.08.2024. Participants were recruited exclusively from the university student population because the original goal of the study was to examine the relationship between childhood trauma and academic success. Of n = 951 subjects who accessed the online questionnaire via the QR code on the promotional materials, n = 638 subjects completed the study. Due to missing values, 14 participants were excluded. The final sample comprised n = 624 participants aged 18–44 years (*M* = 23.56, *SD* = 3.92), including 182 males (29.17%), 429 females (68.75%), and 13 individuals (2.03%) who identified as non-binary. Participants reported a mean emotional abuse score in the CTQ of *M* = 10.49 (*SD* = 4.66), of social devaluation in the SODEQ of *M* = 35.10 (*SD* = 14.72), anxiety and depression in the PHQ-4 of *M* = 4.62 (*SD* = 3.17), and loneliness in the ULS-6 of *M* = 12.76 (*SD* = 4.96).

The Breakup Study: Data for this study was collected between 22.02.2023 and 15.06.2023. Participants were recruited from the general adult population, with invitations posted both on a university student participation portal and on public social media, resulting in a mixed sample of students and non-students. All participants reported the breakup of an intimate relationship within the past 12 months. N = 252 individuals initiated the survey, of which 180 individuals gave consent for their data to be collected and analyzed. Complete data was available for n = 79 participants (79.7% female, 17.7% male, 2.5% non-binary) ranging in age from 20 to 55 years (M = 30.47, SD = 8.63). Participants reported a relationship duration between one and 360 months (M = 62.71; SD = 78.85). Regarding the type of separation, 17.7% of participants reported an amicable separation, 41.8% were abandoned by their partner, 35.4% actively left their partner, and 5.1% reported other scenarios, including “open relationship,” “situational relationship,” or “manipulated into leaving”. Participants reported a mean value of social devaluation in the SODEQ of *M* = 21.78 (*SD* = 14.61), depression in the PHQ-9 of *M* = 10.32 (SD = 6.08), grief in the ICG of *M* = 23.81 (*SD* = 15.44), and social anxiety of *M* = 3.85 (*SD* = 2.82). Due to the limited number of participants in the Breakup Study, the individual analyses include participants who provided sufficient data for the specific analysis even though they skipped questions or questionnaires relevant to other analyses. The specific number of participants is reported for each analysis in the Results section.

The Migration Study: The data for this study was collected between 19.02.2023 and 30.03.2023 and was made available for the scientific analysis at 15.03.2024. This study analyzes data from a community-based sample of N = 511 adult refugees living in four refugee camps (Darashakran, Basirma, Kawargosk, and Qushtapa) in the Kurdistan Region of Iraq (KRI). These four camps are home to approximately 30,000 refugees and asylum seekers of Syrian nationality who have fled the war that resulted from the Syrian Revolution in March 2011. The refugees live in self-built houses ranging from 55 to 100 m^2^. The camps are divided into residential areas and small market areas. The camps are fenced, with a security checkpoint at the entrance. Inside the camp, there is a security unit, a primary health center, schools (both primary and lower secondary), and some aid organizations providing various services. Leaving the camp requires permission from the camp management, as does bringing in new appliances or having overnight visitors. Participants were recruited randomly within the camp. Enumerators made door-to-door visits to randomly selected households within the camps. A single randomly selected adult participant was asked if they would be willing to participate in an interview. The mean age of the selected participants was M = 40.0 years (SD = 12.5), 59% of the sample reported their gender as female, 40% as male, and two individuals did not report their gender identity. The majority of participants were married (80%), and households reported a median monthly income of 200,000 Iraqi dinars, or approximately US$150. Participants reported a mean value of 3.08 social stressors (SD = 1.12), mean social devaluation in the SODEQ was *M* = 22.76 (*SD* = 15.61), and mean depression in the PHQ-9 was *M* = 9.71 (SD = 5.23).

## Measures

### Social devaluation questionnaire

Item Selection. The development of the Social Devaluation Questionnaire (SODEQ) was guided by the theoretical framework of peritraumatic defense reactions described by Neuner [12] and related literature on acute stress responses to physical and social threats [25,30,32,56–59]. While both types of threat activate overlapping neural circuits, including the amygdala and ventral prefrontal cortex [60], research indicates that threats to social status trigger particularly intense and specific emotional, cognitive, and physiological responses [27,61,62]. In social species such as humans, survival and reproduction depend not only on group membership but also on one’s position within a social hierarchy, which regulates access to resources and mating opportunities [63]. Acute threats to status, such as social defeat or degradation, mirror ritualized dominance–submission encounters seen across vertebrates. These encounters often begin with active resistance or fight/flight mobilization but, when defeat is inevitable, shift into a demobilized submissive posture, reduced locomotion, and lowered physiological activity [64,65]. In humans, such defeat states are accompanied by appraisals of inferiority, inadequacy, and loss of social value, as well as behavioral inhibition and low affect [27,66].

The affective core of acute status loss includes emotions such as shame, humiliation, and degradation. Shame reflects an internalized appraisal of diminished worth and motivates withdrawal or appeasement to avoid further harm [25,67]. Humiliation and degradation add the interpersonal dimension of being lowered in rank through the actions of another, often in a way that is public and inescapable. Although exclusion can also provoke intense stress responses [29], in the context of social devaluation it is conceptualized less as a rupture of affiliation and more as a visible sign and consequence of loss of status within the social hierarchy.

On the background of this theory-based selection of indicators, the aim in item formulation was to provide verbally accessible descriptions of each component of the acute status-loss response that are grounded in well-documented biological processes and thus likely to be valid across cultures. This approach increases the likelihood that respondents from diverse linguistic and cultural contexts can recognize and endorse the described experiences, even without specialized psychological vocabulary. The resulting SODEQ covers affective, cognitive, behavioral, and physiological manifestations of social devaluation. Affective items capture shame, humiliation, and degradation. Appraisal items, introduced with “I had the impression that…,” target perceptions of defeat, damaged honor, self-doubt, and loss of self-worth. These appraisals are closely tied to status loss because they reflect the internalized cognitive representation of one’s diminished position in the social hierarchy, a perception that one’s reputation, honor, and standing have been undermined, and that one’s capacity to compete or maintain status has been compromised. Such appraisals are central to the psychological experience of social defeat and degradation and have been repeatedly documented as predictors of withdrawal, submission, and long-term demoralization following status-threatening events. Behavioral impulse items, introduced with “I would have liked to…” or “I felt the urge to…,” address blocked action tendencies to confront, flee, or withdraw—patterns consistent with the fight/flight-to-submission transition in social defeat. Physiological items reflect both the motor inhibition of tonic immobility (“I was frozen”) or flag reactions (“I felt paralyzed”). Although these two may appear similar, pilot interviews with therapists and clinical researchers confirmed that participants recognized them as distinct experiential states, with “frozen” allowing heightened vigilance and readiness to act, and “paralyzed” to complete motor inhibition and emotional blunting.

*Pre-tests* The instrument was pre-tested in an interview format by therapists and clinical researchers with healthy participants and patients in different settings, including patients in an outpatient clinic specializing in severe cases of PTSD, mainly following child abuse, and refugees who had fled from war-affected areas to Germany or regions close to their home countries. The pre-tests involved brief discussions between interviewers and respondents about the content and wording of the items, with the aim of identifying difficulties in understanding the items of the scale. In order to test a more direct reflection of peri-traumatic experiences, the pre-tests also included the administration of the instrument immediately after exposure to various types of traumatic memories during individual Narrative Exposure Therapy sessions. In general, interviewers and therapists reported that respondents could understand the items and that the content of the instrument was perceived as meaningful and relevant by most respondents, regardless of the type of stressful event to which the instrument was related. As a result, most items remained unchanged throughout this process. Although some respondents had difficulty identifying the semantic difference between the two items covering physiological responses (paralysis and numbness), we decided to retain these two items because they assessed a theoretically important different semantic nuance, with paralysis having a stronger motor connotation and numbness having a stronger emotional connotation.

The final version of SODEQ consisted of 14 items, each item formulated as a self-statement. To ensure compatibility with a standard instrument for assessing reactions to stressful events, the Peritraumatic Distress Inventory [3], which may be used to complement the SODEQ, the response format was also constructed as a five-point scale ranging from 0 to 4, with identical anchors 0 = “not at all”, 1 = “a little bit”, 2 = “moderately”, 3 = “quite a bit”, and 4 = “extremely “. Due to a technical error, the “quite a bit” category was omitted from the Peer Victimization Study, limiting the scoring to four categories instead of five. The numerical values were kept the same to maintain comparability with the other applications. Because the SODEQ assesses the affective response to a specific event, condition, or time period, the SODEQ requires that a reference event or condition be pre-defined in the instructions. Based on the assumption that social devaluation can occur in relation to a wide range of events and conditions, and may also represent a generalized response to a wider range of similar situations, the SODEQ has few requirements for the specification of the index event in specific studies. The SODEQ can be used for the retrospective assessment of both experiences and permanent conditions. For the assessment of experiences, the index event should be specified, followed by the instruction “Please answer the following questions to see if they applied to the time during the event or shortly after”, with items formulated in the past tense. For subjects who are currently experiencing a stressful condition, the SODEQ items may refer to specifics of the situation, e.g., “as a refugee...” with items phrased in the present tense. The SODEQ was originally developed in German and translated into English and Arabic by clinical experts familiar with the concept of social devaluation, including a standard procedure of instrument translation based on translation and blinded back-translation. For the purpose of this validation study, the Arabic translation of the SODEQ was used in the Post-Migration study and the German original was used in the other surveys. In contrast to single-event trauma research, social traumas such as peer victimization, abandonment, emotional abuse, and refugee discrimination typically consist of repeated and multifaceted incidents rather than a single episode. To capture this reality, participants in the Migration Study were asked to respond with reference to their status of being a refugee (“As a refugee…”). Pilot testing in the camps confirmed that this framing was well understood, as respondents spontaneously associated the refugee condition with the recurrent humiliations, restrictions, and experiences of discrimination that shape daily life in the camps. This status-based framing thus served as an appropriate reference condition for eliciting the targeted affective responses. Within the different surveys included in this study, the SODEQ was related to different types of events. In the Emotional Abuse study, the SODEQ was related to the emotional abuse items assessed on that particular instrument; in the Peer Victimization study, respondents were asked which of the events on the Peer Victimization Questionnaire was the most stressful, and the SODEQ was related to that event. In the Breakup Study, the SODEQ was related to the separation experience.

### Instruments to assess event severity

Emotional abuse was assessed using the emotional abuse subscale of the German version [68] of the short form of the Childhood Trauma Questionnaire [69]. This scale includes five items related to emotionally abusive events or perceptions of being abused by parents in childhood, which are rated on a five-point scale ranging from 1 = “not at all” to 5 = “very often”. Cronbach’s α in this study *α* = .88. Peer victimization was assessed using the German language Adverse Social Experiences Questionnaire (Fragebogen für belastende Lebenserfahrungen, FBS) [70]. This self-report questionnaire consists of 22 items describing aversive social situations in a peer context such as rejection, exclusion, being laughed at, insulted, and teased by peers (e.g., “I was excluded from games or activities by other children or adolescents,” “I was laughed at in the presence of other children”). For each situation, respondents were asked whether they had experienced the situation during childhood (ages 6–12) or adolescence (ages 13–18). The total score was calculated as the sum of “yes” responses across both age periods and ranged from 0 to 44. The FBS total score showed satisfactory stability over a 20-month period (r = 0.89) and reached a Cronbach’s α of.89 in this study. Separation: Participants were asked to provide information about the separation scenario by classifying their separation into one of four types (being abandoned by their partner, actively abandoning their partner, experiencing a consensual separation, or experiencing another scenario). Separation was coded as abandonment (1) if they were abandoned by their partner, and as no abandonment (0) for all other separation scenarios. Post-migration stress: In the context of this survey, we had developed a specific scale to assess social stressors associated with the refugee experience. We had decided not to rely on standard scales of post-migration stress, such as the Post-Migration Living Difficulty Checklist (PMLD) [71], because these measures often conflate the severity of events with subjective reactions to stressors by asking how “problematic” an event or condition was for them. To provide a more limited estimate of the severity of post-migration stress, we simplified the scale by asking respondents in a yes/no format whether they experienced certain types of stressors in exile. We asked about six social stressors that were commonly reported by refugees in the camp in pilot interviews conducted with refugees in preparation for this survey, i.e., having family members left behind, having family members flee to Europe, experiencing discrimination as a refugee, experiencing lack of job opportunities as a refugee, experiencing the loss of a close person, and experiencing lack of education. The severity of post migration stress was estimated as the sum of items responded with “yes”.

### Mental health

Depression and Anxiety: In the Emotional Abuse Study, mental health symptoms were assessed using the Patient Health Questionnaire (PHQ-4) [72], which assesses symptoms of anxiety and depression with 2 items each. Participants are asked to rate how often they have been affected by symptoms in the past 2 weeks. The response format follows a 4-point Likert scale ranging from 0 = “not at all” to 3 = “almost every day”. The PHQ-4 has satisfactory psychometric properties in the general population [73] and achieved a Cronbach’s alpha of *α* = .85 in this study. The Patient Health Questionnaire 9 (PHQ-9) was used to assess symptoms of depression in all studies except for the Emotional Abuse study. The PHQ-9 is a highly cited and widely used instrument that rates each of the nine DSM-IV criteria from “0” (not at all) to “3” (almost every day). The score for depression symptoms is the sum of all item scores. In the Migration Study, the validated Arabic version of the PHQ-9 [74] was applied, that had reached an internal consistency of Cronbach’s alpha = 0.86 in the translation. Cronbach’s alpha in the Migration Study was α = .89, *α* =.87 in the Breakup Study and *α* =.87 in the Peer Victimization study.

Loneliness. The modified short form of the validated short form of the UCLA Loneliness Scale, the ULS-6 [75], was used in the Emotional Abuse study. The ULS-6 asks about six characteristics of loneliness (e.g., “I lack companionship”) or its opposite (social inclusion, respective items are reverse coded), using a response format ranging from 1 = “never” to 4 = “often”. The ULS-6 shows promising psychometric properties. The scale achieved a Cronbach’s alpha of *α* = .90 in this study.

Social Phobia: In the Peer Victimization study, symptoms of social phobia were assessed using the Social Phobia Inventory (SPIN, German version [76], a screening instrument for the assessment of social phobia in self-report. The SPIN consists of 17 items from three subscales: social anxiety, avoidance, and physiology. The items are rated on a five-point Likert scale (0 = not at all to 4 = extremely distressed) and refer to symptoms during the past week. The SPIN shows good psychometric properties (Sosic et al., 2008), in this sample Cronbach's alpha was α = .93. In the Breakup Study, social anxiety disorder was assessed using the German version of the three item short version of the SPIN, the Mini Social Phobia Inventory (MINI-SPIN; Wiltink et al., 2017). The items of the short questionnaire for recording symptoms of social anxiety are answered on a five-point rating scale from 0 = ‘not at all’ to 4 = ‘extremely’. The MINI-SPIN showed good internal consistency in the present survey (Cronbachˊ s ɑ = .76).

PTSD symptoms: The German version of the Primary Care Posttraumatic Stress Disorder screening questionnaire (PC-PTSD-5) [77] was used to assess PTSD symptoms. In the PTSD screening questionnaire, respondents can answer “yes” or “no” to five different symptoms of PTSD. In this study, the items relate to the separation situation experienced (e.g., “In the past month, have you had nightmares about the separation, or have you had to think about it when you didn’t want to?”). Participants can receive a score from 0 to 5. The PC-PTSD showed an acceptable internal consistency in the present study (Cronbach’s α = .61).

Complicated Grief. A modified version of the German version of the Inventory of Complicated Grief (ICG-D) [78] was used to assess symptoms of grief in the Breakup Study. The items were adapted to correspond to the loss of a person through separation rather than the loss of a person through death. Responses are recorded on a five-point Likert scale with categories ranging from 0 = “never” to 4 = “always”. The ICG, which measures complicated grief, is analyzed here by adding the scores of all items to a total score. The ICG also showed high internal consistency and had a Cronbach’s alpha of α = .94 in the present study.

## Procedure

### Online surveys

Data for the online surveys were collected using Qualtrics software. The link for the Emotional Abuse study was provided through promotional materials at the university. The links to participate in the Peer Victimization and Breakup studies was distributed via social media and posted on a study participation portal for students at Bielefeld University. In all online surveys, participants were informed about the content and risks of the study in a header page. It was also explained that participation in the study was completely voluntary and anonymous and that the study could be terminated at any time. Subjects could then give their consent to participate in the study. In all studies, participants provided informed consent prior to participation. Capacity to consent was not assessed separately, as all participants were legally adults and were recruited in non-clinical contexts (online surveys or community-based sampling). Demographic data (age, gender, years of education and employment) and the study instruments were then collected. All surveys were approved by the Ethics Committee of Bielefeld University.

The data used in the Migration Study were collected as part of an evaluation of a mental health and psychosocial support program provided by the aid organization Un Ponte Per (UPP). UPP is an international organization funded by the United Nations High Commissioner for Refugees to provide a holistic mental health and psychosocial support services (MHPSS) program in refugee camps in the Erbil governorate of the Kurdistan Region of Iraq (KRI). One of the authors of this study trained the local psychologists of the MHPSS team to conduct this assessment. The 5-day training was conducted in Arabic. The psychologists were made aware of the study tools and conducted simulated interviews through role-playing during the training. Three of the local psychologists had psychology degrees from Syria and the fourth had an engineering degree with extensive experience working as a psychologist in aid organizations inside the camp. All team members had received extensive training from UPP for nearly a decade and worked under the supervision of a clinical supervisor and clinical consultant. Participants were recruited by trained community mobilizers, field coordinators, and caseworkers. Interviews were conducted either in the participant’s home or at one of the UPP MHPSS centers inside the camp. At the beginning of the one-on-one interviews, participants were read a detailed informed consent form that explained the risks and benefits of the study and their right to withdrawal. Participants were also given the opportunity to ask questions. The interview took place only after the participant gave verbal consent. All interviews were conducted in the Syrian dialect of Arabic. All participants who were approached agreed to be interviewed. The study protocol was approved by the Ethics Committee of the University of Bielefeld.

### Analysis

Statistical analyses were performed with the R statistical program (R Core Team, 2024), using the *psych* and *lavaan* packages. Internal consistency was assessed using Cronbach’s alpha.

To test whether social devaluation can be assumed to represent a unified affect rather than to determine its precise dimensional structure, we combined principal component methods for factor retention with exploratory factor analysis. Prior to extraction, the suitability of the data was assessed using the Kaiser–Meyer–Olkin (KMO) measure of sampling adequacy and Bartlett’s test of sphericity. The number of factors to retain was evaluated using multiple criteria, including eigenvalues greater than one, scree plots, parallel analysis, Very Simple Structure (VSS), and Velicer’s Minimum Average Partial (MAP) test. Exploratory factor analyses (EFA) were then conducted using principal axis factoring with oblimin rotation, given the expectation of correlated factors. Principal axis factoring was chosen as it does not assume multivariate normality. For each solution, variance explained, factor correlations, and item loadings were examined. All analyses were conducted in R using the psych package. To determine the relationships between event severity, social devaluation, and indicators of mental health, we performed simple mediation analyses using the *lavaan* package of R. The assumption of normal distribution, homoscedasticity, and linearity were confirmed by visual inspection of the respective plots.

## Results

### Reliability

Table 1 presents the results of the item analysis of the SODEQ across the four surveys. Internal consistencies were high in all samples, indicating that the items coherently measured a common construct. All correlation coefficients were bivariate Pearson correlations, except for the correlations between abandonment and SODEQ, which were point-biserial correlations because abandonment was coded as a dichotomous variable.

**Table 1:** Item characteristics of the SODEQ.

	Emotional Abuse			Peer Victimization			Abandonment			Post-migration Stress		
Item	M (*SD*)	Corrected Item – Total Score Correlation	Item-Event Correlation	M (*SD*)	Corrected Item – Total Score Correlation	Item-Event Correlation	M (*SD*)	Corrected Item – Total Score Correlation	Item-Event Correlation	M (*SD*)	Corrected Item – Total Score Correlation	Item-Event Correlation
I felt ashamed.	2.64 (1.33)	.71	.49	1.79 (.99)	.53	.27	.96 (1.24)	.26	0	1.51 (1.36)	.68	.46
I felt humiliated.	2.69 (1.40)	.84	.58	2.09 (.89)	.64	.30	1.52 (1.48)	.84	.42	1.64 (1.39)	.80	.50
I felt degraded.	2.62 (1.42)	.84	.61	1.88 (.96)	.71	.30	1.34 (1.55)	.81	.41	1.85 (1.40)	.81	.55
I had the impression of experiencing a heavy defeat.	2.53 (1.38)	.77	.54	1.68 (.97)	.57	.31	2.14 (1.47)	78	.52	1.62 (1.37)	.75	.51
I had the impression that my honor would be damaged.	1.93 (1.14)	.63	.39	1.21 (1.03)	.51	.18	1.01 (1.25)	.51	.33	0.91 (1.30)	.61	.34
I had the impression of being devalued.	2.54 (1.43)	.81	.62	1.86 (.96)	.65	.32	1.58 (1.56)	.80	.54	1.84 (1.39)	.79	.56
I doubted myself.	3.12 (1.49)	.71	.50	2.26 (.92)	.50	.38	2.17 (1.39)	.65	.38	1.25 (1.32)	.70	.41
I had the impression of having been rejected.	2.75 (1.47)	.74	.55	2.17 (.91)	.46	.26	2.19 (1.58)	.76	.62	2.04 (1.50)	.70	.57
I had the impression of being excluded.	2.32 (1.43)	.64	.50	2.08 (1.04)	.45	.34	1.67 (1.40)	.78	.51	2.04 (1.47)	.62	.52
I would have liked to disappear into the ground.	2.46 (1.51)	.76	.51	1.89 (1.04)	.61	.25	1.20 (1.45)	.61	.32	1.22 (1.45)	.74	.40
I felt the urge to run away.	2.60 (1.56)	.73	.56	1.67 (1.11)	.63	.28	1.66 (1.40)	.54	.27	1.66 (1.57)	.71	.37
I would have liked to yell at someone.	2.62 (1.57)	.48	.40	.98 (1.07)	.18	.21	1.35 (1.58)	.46	.39	1.37 (1.51)	.47	.40
I was frozen.	2.16 (1.41)	.71	.49	1.24 (1.11)	.57	.25	1.45 (1.45)	.70	.43	1.89 (1.45)	.66	.47
I was paralyzed.	2.11 (1.38)	.72	.52	1.14 (1.06)	.57	.24	1.48 (1.44)	.71	.45	1.90 (1.51)	.67	.51
Cronbach’s alpha	.94			.87			.93			.94		

Note.: All correlations are Pearson correlations, except for the Abandonment Study where correlations are point-biserial correlations since the event is coded dichotomously according to separation type (being left versus other types of separation). Abandonment Study analysis is based on data from n = 83 participants.

### Factor analysis

To test whether the SODEQ items consistently reflected a unified underlying construct across samples, principal component and exploratory factor analyses were conducted for three analytic sample (Migration Study, Emotional Abuse Study, and Peer Victimization Study). Prior to factor extraction, the suitability of the data was examined. In all three samples, the Kaiser–Meyer–Olkin (KMO) measure indicated sampling adequacy ranging from .85 (peer victimization study) to .93 (migration study), all considered very good. Bartlett’s test of sphericity was significant in each case (all ps < .001), confirming sufficient intercorrelations among items.

In the Migration Study, initial eigenvalues (first four: 8.54, 1.37, 0.82, 0.69) indicated two components above unity. Parallel analysis supported retaining four factors, whereas VSS and MAP pointed to one. A four-factor EFA explained 59% of the variance, interfactor correlations ranged r = .49–.68 (M = .59). In the Emotional Abuse study, eigenvalues indicated two factors above unity (7.78, 1.17); parallel analysis suggested four to five factors, while VSS and MAP indicated one factor. A five-factor EFA explained 58% of the variance; interfactor correlations ranged from r = .33 to.64 (M = .52). In the Peer Victimization study, eigenvalues revealed three factors above unity (5.72, 1.54, 1.17), parallel analysis suggested five factors; MAP and VSS indicated one. A five-factor EFA explained 52% of the variance, interfactor correlations ranged from r = .22 to.56 (M = .41). In sum, across studies, factor retention criteria consistently indicated a strong general factor alongside multiple correlated subfactors (interfactor rs = .22-.68).

### Construct validity and mediation analyses

**Fig 1:**
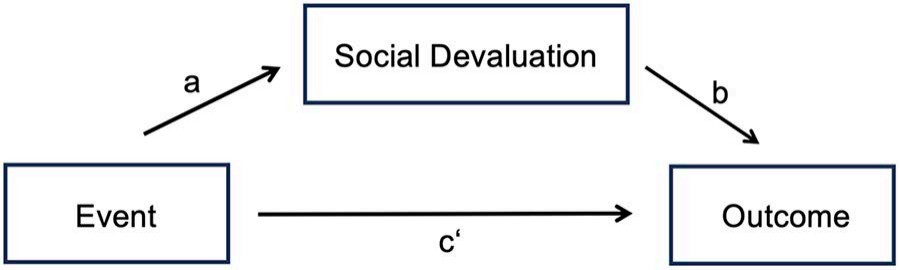
Illustration of Simple Mediation Model.

To examine the proposed mediational role of social devaluation, we tested simple mediation models in which event severity predicted mental health outcomes both directly and indirectly via social devaluation. The assumption checks for normal distribution, homoscedasticity, and linearity showed no substantial deviations from the requirements of mediation analysis. Fig 1 illustrates the conceptual model, showing the direct paths from event severity to the outcome variables (c’), the path from event severity to social devaluation (a), and the path from social devaluation to the outcome variables (b).

Table 2 summarizes the mediation results for each event type and outcome. For example, in the Emotional Abuse Study, emotional abuse severity was strongly associated with social devaluation (path a = .70, p < .001), and social devaluation was in turn associated with higher depression scores. The indirect effect (a*b = .19, p < .001) indicates that a substantial proportion of the relationship between emotional abuse and depression was mediated by social devaluation, even when the direct effect remained significant. Comparable patterns were found for other outcomes and event types, with variations in the size and significance of the indirect effects. For instance, in the Peer Victimization Study, social devaluation significantly mediated the association between peer victimization and both depression and social anxiety. In the Abandonment Study, indirect effects via social devaluation were observed for multiple outcomes, including depression, grief, and social anxiety. Finally, in the Post-Migration Stress Study, the indirect effect through social devaluation was significant for depression, indicating that loss of status and perceived devaluation were key pathways linking post-migration stress to poor mental health.

**Table 2:** Effects of Event Types on Outcomes with Social Devaluation as Mediator.

	Social Devaluation	Depression		Loneliness		Social Anxiety		PTSD Symptoms		Grief	
	Bivariate (a)	Direct (c‘)	Indirect (a*b)	Direct (c‘)	Indirect (a*b)	Direct (c‘)	Indirect (a*b)	Direct (c‘)	Indirect (a*b)	Direct (c‘)	Indirect (a*b)
Emotional Abuse	.70***	.27***	.19***	.21***	.23***						
Peer Victimization	.44***	.42***	.07**			.22***	.13***				
Abandonment	.54***	.06	.32***			-.16	.35***	.11	.27***	.03	.32***
Post-migration Stress	.60***	.01	.26***								

Note: Abandonment Study analyses are based on n = 83 respondents for depression and grief, n = 91 participants for PTSD Symptoms and n = 88 subjects for social anxiety. In all studies depression was assessed with the PHQ-9, except for the Emotional Abuse study that used the PHQ-4 which includes also symptoms of anxiety.

## Discussion

In this study, we hypothesized that social devaluation is a common affective-cognitive response to social stressors. We developed the Social Devaluation Questionnaire, the SODEQ, to assess this state of social devaluation and tested this instrument in relation to three common social stressors as well as in the specific context of refugees living in the Kurdistan Region of Iraq. We found initial evidence for the psychometric quality of the instrument and its consistency with theoretical assumptions.

The SODEQ showed high internal consistency reaching excellent values between (α = .87-.94; see Table 1). These values are based on high intercorrelations of the SODEQ items, with most item-total correlations exceeding r = .45 (see Table 1). This high internal consistency is not self-evident, since the SODEQ assesses the concept of social devaluation as a multimodal affect based on different indicators, such as emotions, appraisals, behavioral tendencies, and physiological reactions. The internal consistency of the SODEQ is not lower than that of other instruments assessing peritraumatic experiences, such as mental defeat (alpha = .90; [7] or dissociation (alpha = .74; [79], even though these instruments are limited to the assessment of single aspects, either appraisals or abnormal experiences. The high internal consistency of the SODEQ is not only an indicator of the reliability of the instrument, but is also consistent with the theoretical assumption that social devaluation is a unified affect. To further examine this assumption, we combined factor-retention heuristics (eigen >1, scree, parallel analysis, VSS, MAP) with exploratory factor analyses across three study samples. Very Simple Structure and Velicer’s MAP test consistently indicated a dominant single factor, while parallel analysis suggested several subfactors. These subfactors corresponded to clusters of items but were always intercorrelated, confirming that they represent facets of the same latent construct. In our study, differences in the number and configuration of subfactors across studies are difficult to interpret, as the samples varied in event type and cultural context. A systematic investigation of measurement invariance across populations will therefore require future studies with larger and more comparable samples. Together, the consistently high reliability and the presence of a strong general factor across diverse samples support the conclusion that social devaluation is best modeled as a unified construct with correlated facets. Other instruments that assess the intensity of negative experiences during stressful events across different types of negative affect, such as the Peritraumatic Distress Inventory, have achieved lower values of internal consistency (α = .75), with no confirmation of factor structure [3].

The validity of the SODEQ is indicated by a meaningful correlation of the score with indicators of the severity of social stressors. Social devaluation was highly related to the severity of emotional abuse and peer victimization reported by the respondents in validated and well-established scales. Social devaluation is strongly associated with emotional abuse because emotional abuse ultimately conveys the message that one is essentially worthless to one’s parents [38]. The same is true for peer victimization, that consists of repeated rejections, exclusions and relational violation [17] and is related with a loss of status and popularity among schoolmates [80]. In the case of intimate partner breakups, it is not surprising that being abandoned by someone is more distressing than a consensual breakup or abandonment, and that this additional distress is related to a perceived devaluation. There is surprisingly little research on the mental health consequences of abandonment by a partner, and our study findings indicate that this event increases the probability of mental health impairments and involves the perception of being devalued. Our findings also confirmed that the refugees’ experiences of post-migration stressors are associated with the perception of rejection and devaluation [49], which, in turn, contribute to psychological disorders.

Social devaluation, as measured by the SODEQ, was related not only to indicators of event severity, but also to a wide range of mental health outcomes, i.e., depression, social anxiety, loneliness, grief, and PTSD symptoms. For different types of peritraumatic reactions, including mental defeat [81], dissociation [4], and various types of negative emotions [1], it is well documented that the quality and intensity of experiences during traumatic events predict the resulting psychological symptoms. The assumption behind the concept of devaluation is that trans-diagnostic trauma-related psychopathology results, in part, from memory representations of cumulative social devaluation experiences [12]. Consistent with this theory, we found that social devaluation mediated the relationship between event severity and psychopathology across different types of events. This finding is consistent with the recent emphasis on events involving status loss in explaining social anxiety, and extends these findings to other types of symptoms.

Although we have found initial evidence for the psychometric soundness of the SODEQ, it is clear that more systematic research is needed to prove the validity of the instrument and the construct of social devaluation. In line with the theoretical assumption, the ultimate criterion is the predictive validity of the SODEQ, which needs to be determined in a longitudinal study. All mediation models presented in this study are based on cross-sectional data, which leaves room for alternative causal explanations. It remains to be shown to what extent social devaluation, as assessed by the SODEQ during or immediately after a stressful event, can predict subsequent psychopathology at a later point in time. The second question that remains to be answered is the specificity of social devaluation. As long as only a single construct is used to assess the affective response to an event, it is likely that this indicator functions in part as a general proxy for the perceived severity of the event and carries along variance related to other emotions such as helplessness and fear. To determine the specific role of social devaluation, it will be important to examine the extent to which the SODEQ predicts psychopathology beyond other well-documented types of peritraumatic affect, such as helplessness, dissociation, and appraisals of mental defeat. The generalizability of our findings is limited because recruitment for our online surveys was uncontrolled and resulted in samples that were far from representative, with an over representation of female participants in the online surveys. However, in the Migration study, we have a first indication that the SODEQ can be applied across contexts and cultures and may be a useful tool to explain part of the relationship between societal hardship and mental health symptoms. However, the cultural and gendered context of social devaluation requires further consideration. Concepts such as shame, humiliation, and degradation may vary in their meaning, salience, and expression across cultural settings, and gender roles in particular can shape how these emotions are experienced and processed. Future research should therefore investigate how socialization, norms, and historical context influence the way social threats and status loss are perceived and expressed. Likewise, our study focused exclusively on adverse and stressful experiences. We did not assess protective or resilience-promoting factors that might buffer the negative impact of social devaluation, even though these may be highly relevant in understanding long-term outcomes. Furthermore, this study is limited by a technical error in the administration of the SODEQ in the Peer Victimization Study, which resulted in the omission of the ‘quite a bit’ response category. This error likely restricted variance and thus may have attenuated associations with external variables. Importantly, this limitation operates against our hypotheses rather than in favor of them, suggesting that the significant associations observed in this sample are conservative estimates rather than inflated effects.

Taken together, despite some limitations, our study indicates that social devaluation as measured with the SODEQ is a promising candidate for the study of psychopathological mechanisms after intense traumatic stress. In future research, the SODEQ might complement existing instruments on peritraumatic distress with a theoretically founded conceptualization of a defensive reaction to severe stress. With a focus on social stress, this instrument might be useful to extend the current focus on traumatic stress research, that is mainly based on events that fulfil the A-criterion of PTSD as physically threatening, by a stronger focus on social threats.

## Supporting information

S1 Questionnaire SODEQ Questionnaire. (PDF)

S1 Checklist Inclusivity in Global Research Questionnaire. (DOCX)
