# Supplementary material for: Measuring the experience of social trauma: Development and evaluation of the Social Devaluation Questionnaire
Source: PLOS Ment Health. 2025 Oct 27;2(10):e0000450. doi: 10.1371/journal.pmen.0000450 (PMC12798448; doi:10.1371/journal.pmen.0000450)
Supplement: S1 Questionnaire — (PDF) [file pmen.0000450.s001.pdf]

Social Devaluation Questionnaire (SODEQ)

Frank Neuner, Bielefeld University, 24.01.2022

Application

As a first step, the SODEQ requires the identification of an *index event* (or *index condition*) that it refers to. In the context of a retrospective assessment this index event may be derived as worst event or a constellation of biographical events from an event checklist. Alternatively, the index event may be represented by a default event presented in an experimental provocation or as a target event shared by a population (e.g. child birth, job loss).

Social Devaluation Questionnnaire (German version)

(Flexible Bestimmung des Index-Ereignisses oder der Bedingung)

Ereignis_______________

Bitte beantworten sie die folgenden Fragen daraufhin, ob sie für die Zeit während des Ereignisses oder kurz danach zutrafen.

**Nr**	**Item**	**über-haupt nicht (0)**	**ein wenig (1)**	**ziem-lich (2)**	**stark (3)**	**sehr stark (4)**
1	Ich habe mich geschämt.					
2	Ich fühlte mich gedemütigt.					
3	Ich fühlte mich erniedrigt.					
4	Ich hatte den Eindruck, eine schwere Niederlage zu erleben.					
5	Ich hatte den Eindruck, dass meine Ehre beschädigt wird.					
6	Ich hatte den Eindruck, entwertet zu werden					
7	Ich zweifelte an mir selbst					
8	Ich hatte den Eindruck, zurückgewiesen worden zu sein					
9	Ich hatte den Eindruck, ausgeschlossen zu werden					
10	Ich wäre am liebsten im Erdboden verschwunden					
11	Ich spürte den Drang, davonzulaufen					
12	Ich hätte am liebsten jemanden angeschrien					
13	Ich war wie eingefroren					
14	Ich war wie gelähmt					

Social Devaluation Questionnnaire (English version)

(Flexible determination of the index event or condition)

Event______________

Please answer the following questions to see if they applied to the *time during the event or shortly after*.

Nr	Item	Not at all (0)	A little bit (1)	Mode-rately (2)	Quite a bit (3)	Ex-tremely (4)
1	I felt ashamed.					
2	I felt humiliated.					
3	I felt degraded.					
4	I had the impression of experiencing a heavy defeat.					
5	I had the impression that my honor would be damaged.					
6	I had the impression of being devalued.					
7	I doubted myself.					
8	I had the impression of having been rejected.					
9	I had the impression of being excluded.					
10	I would have liked to disappear into the ground.					
11	I felt the urge to run away.					
12	I would have liked to yell at someone.					
13	I was frozen.					
14	I was paralyzed.					
