# Supplementary material for: Measuring the experience of social trauma: Development and evaluation of the Social Devaluation Questionnaire
Source: PLOS Ment Health. 2025 Oct 27;2(10):e0000450. doi: 10.1371/journal.pmen.0000450 (PMC12798448; doi:10.1371/journal.pmen.0000450)
Supplement: S1 Checklist — (DOCX) [file pmen.0000450.s002.docx]

Inclusivity in global research

PLOS’ policy on inclusivity in global research aims to improve transparency in the reporting of research performed outside of researchers’ own country or community and ensures that PLOS publications reporting global research adhere to high standards for research ethics and authorship. Authors of relevant research articles may be asked to complete the questionnaire below, which outlines ethical, cultural, and scientific considerations specific to inclusivity in global research. This questionnaire may be requested when researchers have travelled to a different country to conduct research, if research uses samples collected in another country, research with Indigenous populations or their lands, or if research is on cultural artefacts. Researchers travelling to another country solely to use laboratory equipment will not normally be required to complete the questionnaire. However, the questionnaire can be requested at the journal’s discretion for any submission – if you have been requested to complete this questionnaire by the PLOS journal you submitted to, please do so.

Please complete the questionnaire below and include this as a Supporting Information file with your manuscript. Note that if your paper is accepted for publication, this checklist will be published with your article in the supporting information files. Please ensure that you reference the checklist in the main body of your manuscript. We suggest adding a subsection ‘Inclusivity in global research’ to your Methods section and adding the following sentence: “Additional information regarding the ethical, cultural, and scientific considerations specific to inclusivity in global research is included in the Supporting Information (SX Checklist)”

The questions have been designed to be applicable to a wide range of study types, and there are subsections for both human subjects research and non-human subjects research. If any of the questions are not relevant to your research please mark them as “N/A” as appropriate.

**Ethical considerations, permits and authorship**

*This section is applicable to all research types.*

Provide details as to who granted permissions and/or consent for the study to take place in the Methods section of your manuscript. This should include the names of **all** ethics boards, governmental organizations, community leaders or other bodies that provided approval for the study. If individuals provided approval refer to these people by their role or title but do not list their name(s).

Reported on page number: The study protocol was approved by the Bielefed University Board of Ethics – approval number 2023-052 of 02.02.2024.

If there were any deviations from the study protocol after approval was obtained please provide details of these changes in the Methods section of your manuscript.
Did this study involve local collaborators that are residents of the country where the research was conducted or members of the community studied? If you do not have any authors from said communities, please provide an explanation for this below.

Reported on page number: none.

Yes.

Everyone listed as an author should meet PLOS’ criteria for authorship and all individuals who meet these criteria should be included in the author byline, rather than the acknowledgements. For further information please see the journal’s Authorship Policy.

**Human subjects research (e.g. health research, medical research, cross-cultural psychology)**

Did you obtain written informed consent from a representative of the local community or region before the research took place? How did you establish who speaks for the community? Details of written informed consent obtained from study participants should be reported separately in the Methods section of your manuscript.

We obtained **verbal informed consent** from all participants using a standardized consent form administered by Un Ponte Per (UPP) field staff in the camps. The form explained the study purpose and procedures (≈60-minute interview), emphasized **voluntary participation** (right to skip any question and withdraw at any time without consequences), clarified **no government affiliation** of the NGO, and described **potential emotional discomfort**from recalling past experiences along with **referral pathways** to existing camp NGOs if needed. We assured **confidentiality** and stated that de-identified data would be used in scientific publications. We disclosed that there were **no financial or other direct benefits** for participation. Participants could opt in separately to **referral to services**. The form also informed participants of a **time-limited right to request deletion** of their data for up to**6 weeks after the last interview (scheduled 31.03.2023)** via the local UPP center (with operational details such as tent/house number used solely to locate records). Participants could ask questions at any time, and the interviewer documented consent on the form

How did members of the local community provide input on the aims of the research investigation, its methodology, and its anticipated outcome(s)?

Local staff from Un Ponte Per (UPP) were actively consulted during the design phase and training on the interview tools. Their feedback was invaluable in refining the study, particularly with respect to the **language and cultural comprehensibility of the scales,** and in ensuring that the instruments were understandable and appropriate for participants. In addition, their **input on logistical and practical aspects of study execution** helped adapt procedures to the camp setting and facilitated smooth implementation. This collaborative process ensured that the research aims, methodology, and anticipated outcomes were informed by local expertise and community context. Importantly, this collaborative process fostered **shared ownership of the project,** aligning the research aims and methodology with local priorities and sensitivities. By incorporating their expertise, the study was grounded in the lived realities of the community and better positioned to generate findings that are both scientifically meaningful and socially relevant.

When engaging with the local community, how did you ensure that the informed consent documents and other materials could be understood by local stakeholders?

We worked closely with local Un Ponte Per (UPP) staff to ensure that all informed consent documents and study materials were **translated into the local language** and presented in **clear, contexually appropriate wording**. The forms were reviewed by local staff for accuracy and comprehensibility, and interviewers were trained to deliver the information orally and answer questions in accessible terms. Particular care was taken to explain key concepts, such as confidentiality, voluntary participation, and referral pathways, in ways that were **aligned with local cultural understandings.** This process helped ensure that participants and community stakeholders could meaningfully understand and evaluate their involvement in the study

Will the findings of the research be made available in an understandable format to stakeholders in the community where the study was conducted (e.g. via a presentation, summary report, copies of publications, etc.)? Please provide details of how this will be achieved.

Yes. Findings were shared with community stakeholders and humanitarian partners in the form of a**summary report tailored to advocacy purposes**, which emphasized clear language, visual presentation of results, and practical implications. This report was used to advocate for the continuation of **mental health and psychosocial support (MHPSS) services** in the camps and was submitted to UNHCR and other relevant actors. In addition, debriefing sessions were conducted with local UPP staff, who continue to communicate findings back to the community in accessible ways through their ongoing service delivery. Future peer-reviewed publications will also be shared in summary form with local partners to ensure the results remain accessible to non-specialist stakeholders.

**Non-human subjects research using specimens/ animals collected as part of the study, or those housed in archival collections. Examples include archaeology, paleontology, botany and zoology.**

Did the permission you obtained from a local authority to perform the study include an agreement on access to outputs and benefit sharing? This may include procedures to enable fair distribution of the benefits and resources arising from the research performed. Please include any details of Prior Informed Consent and Benefit Sharing Agreements obtained. These may be required by field-specific regulations, for example the Convention on Biological Diversity (CBD) and the associated Nagoya Protocol.

If the material used in your study was imported, please A) provide the year it was imported and B) indicate whether permits were obtained to import/export the materials used, C) provide details of any permits obtained. If this information is not available, please indicate this.

If you used archival specimens, please state how the material used in your study was acquired by the institute it is held in and provide details of any permits obtained for the original excavations/ sample collection. If this information is not available, please indicate this.

How was the potential cultural significance of the materials collected in your study to local communities considered in your research design? Were Indigenous peoples and/or local researchers and institutions involved with archaeological excavations / collection of specimens? If so, please provide a description of their involvement.

If your manuscript includes photographs of human remains please indicate whether authors obtained permission from descendants or affiliated cultural communities to do so.
